# Tunable upconversion emission in NaLuF_4_–glass-ceramic fibers doped with Er^3+^ and Yb^3+^

**DOI:** 10.1039/c9ra05182a

**Published:** 2019-10-08

**Authors:** G. Gorni, Jose J. Velázquez, M. Kochanowicz, D. Dorosz, R. Balda, J. Fernández, A. Durán, M. J. Pascual

**Affiliations:** Ceramics and Glass Institute, CSIC Madrid Spain ggorni@icv.csic.es; FunGlass—Centre for Functional and Surface Functionalized Glass, Alexander Dubček University of Trenčín Trenčín Slovakia; Bialystok University of Technology, Faculty of Electrical Engineering Bialystok Poland; AGH University of Science and Technology, Faculty of Materials Science and Ceramics Krakow Poland; Applied Physic Department I, Superior School of Engineering, Basque Country University Bilbao Spain; Materials Physics Center CSIC-UPV/EHU San Sebastian Spain; Donostia International Physics Center San Sebastian Spain

## Abstract

Novel glass-ceramic optical fibers containing NaLuF_4_ nanocrystals doped with 0.5ErF_3_ and 2YbF_3_ (mol%) have been prepared by the rod-in-tube method and controlled crystallization. NaLuF_4_ nanocrystals with a size around 20 nm are obtained after heat treatment at 600 °C. Intense upconverted green and red emissions due to (^2^H_11/2_, ^4^S_3/2_) → ^4^I_15/2_ and ^4^F_9/2_ → ^4^I_15/2_ transitions, respectively, together with a blue emission due to ^2^H_9/2_ → ^4^I_15/2_ transition have been observed under excitation at 980 nm. The intensity of the green and red upconversion bands shows a nearly linear dependence on the excitation power which can be explained by saturation effects in the intermediate energy states and proves that a sensitized energy transfer upconversion process is responsible for the population of the emitting levels of Er^3+^ ions. The upconversion emission color changes from yellow to green by increasing the excitation power density which allows to manipulate the color output of the Er^3+^ emission in the glass-ceramic fibers. The tunable emission color is easily detected with the naked eye. This interesting characteristic makes these glass-ceramic fibers promising materials for photonic applications.

## Abstract 2

Novel glass-ceramic optical fibers containing NaLuF_4_ nanocrystals doped with 0.5ErF_3_ and 2YbF_3_ (mol%) have been prepared by the rod-in-tube method and controlled crystallization.

## Graphical abstract

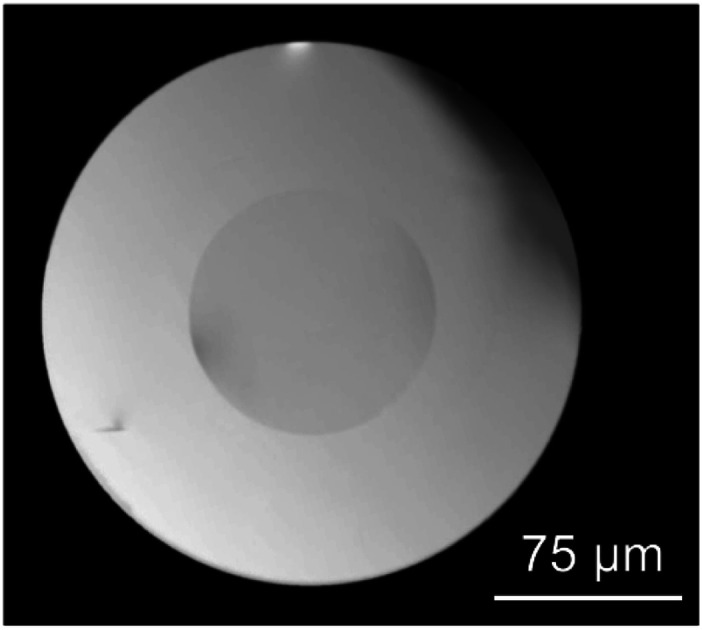

## Introduction

Oxyfluoride glass-ceramics (OxGCs) have received great attention since the preparation of transparent materials in the 1990s.^1^ During the last 7 years, several review papers regarding these materials were published.^2–7^ Higher photoluminescence (PL) efficiencies and better energy-transfer (ET) are usually obtained in OxGCs with respect to glasses due to the controlled crystallization of low phonon-energy fluoride nano-crystals (NCs) that are good hosts for Rare-Earth (RE) ions.^8–18^ Most glass systems are based on aluminosilicate compositions that show good mechanical, thermal and chemical properties together with improved PL features of fluoride NCs doped with RE ions. Most studies were performed on bulk samples but the possibility to prepare GC fibers is also becoming a hot-spot for novel optical materials.

The first studies about transparent GC fibers date back to the end of the 1990s when P. A. Tick indicated four conditions to achieve this goal:^19^ (1) crystal size smaller than 15 nm; (2) interparticle spacing comparable with the crystal size; (3) narrow particle size distribution and (4) absence of clustering. Some years later, Tick *et al.* produced fibers with a core of 5 μm using the double crucible method starting from a Wang and Ohwaki's modified composition in the system SiO_2_–Al_2_O_3_–PbF_2_–CdF_2_–YF_3_.^20^ After the crystallization process at 440–470 °C, a crystalline YF_3_–CdF_2_–PbF_2_ solid solution with an average size ∼6.5 nm was observed in the GC fiber. The authors concluded that low losses, ∼1 dB m^−1^ or less, are possible in such systems in spite of a relatively large refractive index mismatch (0.06). In 2001, Samson *et al.*^21^ described the first efficient GC fiber laser doped with Nd^3+^ using the same glass system and drawing process reported by P. A. Tick.^20^ Similar slope efficiencies ∼30% were obtained for glass and GC fibers, but the presence of NCs embedded within the core of the single-mode optical fiber allowed enhancing the fluorescence and gain spectrum. R. Lisiecki *et al.*^22^ and Augustyn *et al.*^23^ prepared GC fibers starting from the glass composition (mol%) 48SiO_2_–11Al_2_O_3_–7Na_2_O–10CaO–10PbO–11PbF_2_ doped with 3ErF_3_ and co-doped with 1ErF_3_ and 3YbF_3_. The fibers were drawn at 720–740 °C using the rod-in-tube method, and then crystallized at 700 °C. Pb_5_Al_3_F_19_, Er_4_F_2_O_11_Si_3_ and Er_3_FO_10_Si_3_ crystalline phases were obtained with size below 10 nm. The incorporation of RE dopants in the crystal phases was indicated by a narrowing of the emission spectra and longer lifetime of the first excited state of Er^3+^. The authors concluded that such materials may be considered as a promising candidate for Erbium-Doped-Fiber-Amplifier (EDFA) operating within the telecommunication window. C. Koepke *et al.*^24^ prepared fibers with the same composition doped with 0.6YbF_3_ and 0.2ErF_3_ (mol%). The luminescence efficiency of the fiber was two times higher with respect to the untreated fiber. Temperature dependence of Er^3+^ luminescence at 1530 nm was observed by varying the temperature from 5 to 350 K. Such dependence was different for the GC fiber and associated to a decrease of the phonon energy in the Er^3+^ surrounding. In 2012, one of the authors D. Dorosz *et al.*^25^ reported the preparation of Nd^3+^-doped OxGC fibers in the system SiO_2_–Al_2_O_3_–ZnO–Na_2_O–SrF_2_ using the rod-in-tube method. SrF_2_ NCs with size ∼60 nm were observed after heat treatment at 635 °C. After excitation of Nd^3+^ ions at 808 nm only the ^4^F_3/2_–^4^I_11/2_ transition was observed, instead the three emissions ^4^F_3/2_–^4^I_9/2_, ^4^F_3/2_–^4^I_11/2_ and ^4^F_3/2_–^4^I_15/2_ were detected in the bulk GC sample. Moreover, a red-shift of the ^4^F_3/2_–^4^I_11/2_ emission was observed passing from bulk (1060 nm) to fiber (1066 nm) and the authors explained this result by an amplified spontaneous emission occurring in the fiber. Recently, V. K. Krishnaiah *et al.*^26^ described the preparation and properties of Yb-doped single-index GC fibers produced by two methods: (1) by conventional drawing from a glass preform and, (2) by single crucible method (direct-melt process) followed by controlled heat-treatment. The glass system chosen by the authors, a variant of the initial Wang and Ohwaki's composition was: 30SiO_2_–15Al_2_O_3_–27CdF_2_–22PbF_2_–4YF_3_–2YbF_3_ (mol%). The authors observed that by using the second method, better quality GCs were obtained with NCs ∼10 nm homogeneously distributed along the fiber. After crystallization, higher Yb^3+^ PL intensity and quantum yield (0.95) were obtained with respect to the untreated fiber (0.82). Some of the authors obtained LaF_3_-GC fibers doped with Nd^3+^ and demonstrated the possibility to reproduce crystal-like optical features in GC fibers.^27^ An extended structural characterization was carried out to estimate the dopants distribution and the nanostructure of the GC fiber. Other authors obtained fluoride NCs in oxide matrices by the melt-in-tube method using borosilicates cladding and drawing temperatures at which the cladding glass softens but the core is completely melted.^28–33^ For example, Kang *et al.*^28^ obtained NaYF_4_ NCs in borosilicate glasses after heat treatment at 470–500 °C and much better ET from Ho^3+^ to Er^3+^ was observed in the glass-ceramic fibers. However, to the best of our knowledge, this method has not still been applied to aluminosilicate compositions because they require the use of SiO_2_ cladding and extremely high melting temperatures (1800 °C) that can cause strong fluorine loss and diffusion phenomena. Other preparation methods in addition to the GC route were also studied, for example Shahzad *et al.*^34^ prepared PMMA fibers containing LiYF_4_ NCs co-doped with Yb^3+^–Er^3^ for applications as optical sensors.

In this study, we report the preparation and upconversion (UC) emission of novel OxGC fibers based on cubic solid solutions of the type Na_*x*_Lu_2*x*−1_F_7*x*−3_ doped with 0.5ErF_3_ and 2YbF_3_ (mol%).^15^ This crystal phase was selected because it shows efficient UC process.^35–38^

Moreover, the Er^3+^/Yb^3+^ ratio 0.5/2 showed much better UC emission than Er^3+^-single doped glass.^38,39^ The dependence of the upconversion emission on the excitation power intensity is investigated.

## Experimental methods

### Materials preparation

Glasses of composition (mol%) 70SiO_2_–5Al_2_O_3_–2AlF_3_–2Na_2_O–18NaF_3_–3Lu_2_O_3_ doped with 0.5ErF_3_ and 2YbF_3_ were prepared as described elsewhere.^38^ Glass rods were polished and used as core materials for the rod-in-tube method. The drawing process was performed at 1230 °C and the subsequent crystallization at 600 °C for 20 h using a heating rate of 10 °C min^−1^ and a cooling rate of 1 °C min^−1^ to minimize the mechanical stresses between cladding and core. The cross-section of the fibers was observed using a ZEISS Axiophot microscope equipped with a Zeiss AxioCam MR 5 camera and the ZEN 2.3 lite software for calibrating the images.

### Structural and optical characterization

Powder X-Ray-Diffraction (XRD) was performed using the Bruker D8 Advanced diffractometer (*λ* = 1.54056 Å – CuKα1). The powders were milled to a particle size less than 60 μm and both the as drawn fibers and GC fibers were measured. The diffractograms were acquired in the range 10–60° using a step size of 0.02°. The crystal phase was analyzed with the software EVADiffractPLUS. The crystallite size, *ϕ*, was calculated using the Scherrer's equation:^40^1
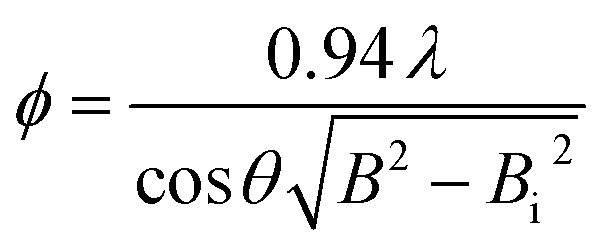
where *θ* is the angle of the diffraction maximum, *B* its full width at half maximum, *B*_i_ the instrumental broadening and *λ* is the wavelength. The *θ* and *B* parameters were obtained by fitting the peaks to pseudo-Voigt functions.

Fourier Transform Infrared Spectroscopy (FTIR) was performed in the range 600–200 cm^−1^ using a Nicolet 6700-Thermo Scientific in ATR configuration. The resolution was set to 4 cm^−1^ and 1024 spectra were acquired for each sample.

High-resolution transmission electron microscopy (HRTEM) was performed on glass and GC fibers using a JEOL 2100 field gun emission TEM with a point resolution of 0.19 nm. Selected area diffraction patterns (SAED) were also acquired. Powder samples were dispersed in ethanol and then few drops deposited onto carbon-coated copper grids and the solvent was removed by drying under UV lamp.

Steady-state emission spectra were recorded by transversely exciting the fibers with a continuous wave (CW) 980 nm semiconductor laser diode. The fluorescence was analyzed with a 0.25 m monochromator, and the signal was detected with a Hamamatsu R636 photomultiplier and finally amplified using a standard lock-in technique.

The transmission losses at 633 nm were determined by using a conventional cut-back (or differential) method.^41^ The input for the power was provided by a He–Ne laser and the transmitted power was measured using a Si detector. Then, the fiber is cut back and the transmitted power measured again. The loss of the fiber in decibels per unit length is obtained from the measured transmitted power ratio of the two measurements.

## Results and discussion

### Glass and GC fibers

**Fig. 1:**
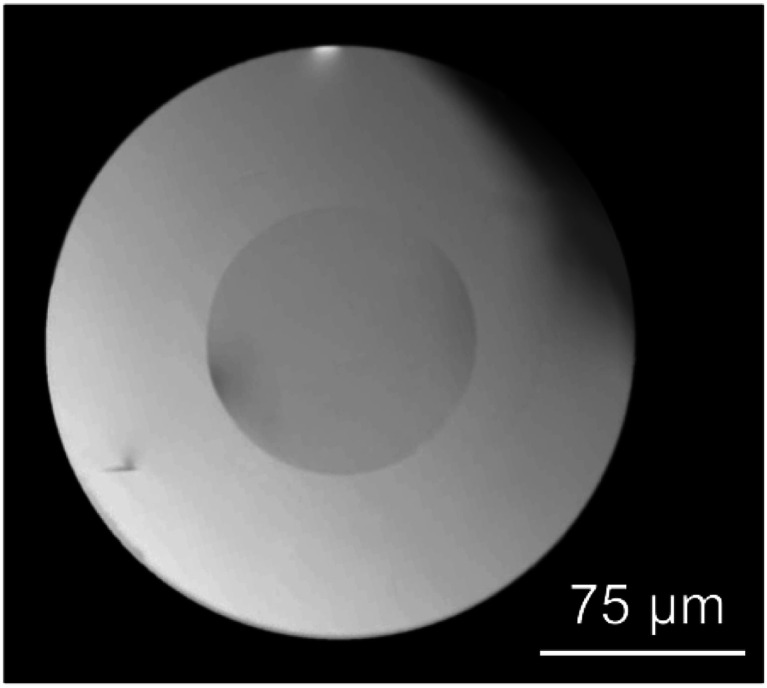
Transversal section of the fiber.

A detailed thermal characterization of the precursor glass was reported previously.^38^ The glass transition temperature *T*_g_ is around 585 °C, the coefficient of thermal expansion (CTE) *α* around 7.5 × 10^−6^ °C^−1^, the refractive index is 1.49 at 588 nm and the log *η* = 4.1 at 1000 °C, being *η* the viscosity in dPa s. DURAN® glass was used as cladding glass, despite *T*_g_ is around 525 °C and CTE is 3.3 × 10^−6^ °C^−1^, due to its quite high working point (log *η* = 4 at 1260 °C, *η* in dPa s) and low refractive index (1.473 at 587.6 nm). A stable process was obtained by drawing the fibers in the range 1100–1230 °C because these temperatures are suitable for the DURAN® cladding glass. However, transparent fibers were only obtained by raising the temperature to 1230 °C. In fact, oxyfluoride compositions tend to crystallize when heated above the *T*_g_ and as known,the crystallization tendency decreases with faster cooling rates. In the standard procedure as the rod-in-tube method, the cooling of the core is slow down by the thermal screening of the cladding glass. This explains why fibers produced with direct-melt are transparent even for drawing at ∼1000 °C while drawing at the same temperature using a cladding glass can cause the spontaneous crystallization of the core. The drawing speed was adjusted to obtain a cladding/core diameter of approx. 225/100 μm as shown in Fig. 1.

### XRD and crystal size

**Fig. 2:**
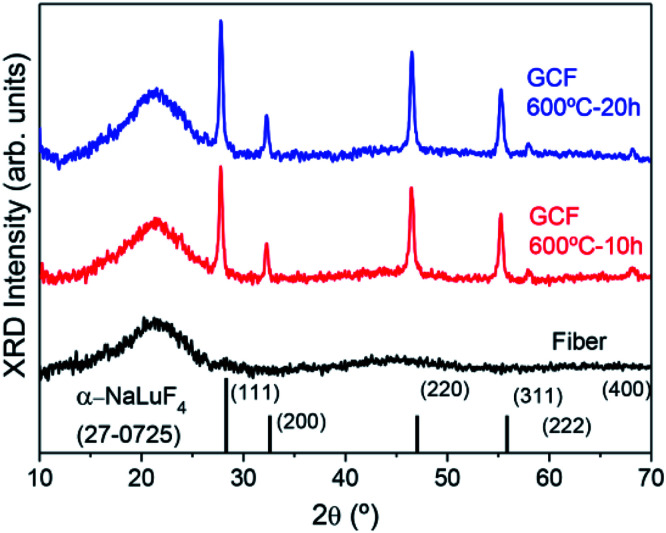
XRD of as made fibers and GC fibers treated at 600 °C for 10 and 20 h.

Fig. 2 shows XRD results of as drawn fibers and GC fibers treated at 600 °C for 10 and 20 h. In the first case, an amorphous pattern is obtained confirming the absence of crystals in the drawn fibers. However, the presence of tiny crystals smaller than 2–3 nm cannot be excluded. For the GC sample, well-defined diffraction maxima are identified and compared to those obtained for the bulk sample treated at the same temperature. It can be concluded that the same cubic solid-solution NaLuF_4_ with fluorite-type structure is obtained. The reference pattern is the one of NaLuF_4_ (JCPDS 27-0725) which has the same cubic structure. The crystal size after heat treatment at 600 °C for 20 h is ∼20 nm, while for bulk GC, treated in the same condition, the crystal size is ∼42 nm.^38^ This phenomenon was well studied in a previous paper.^27^ Due to higher cooling rate of the drawing process, the initial fiber present less pronounced phase separation with respect to a bulk material of the same composition. Since the phase separation zones are the precursor for crystallization, there is a delay in the crystallization process of the fibers. Therefore, for the same heat treatment temperature, smaller crystals are formed in GC fibers with respect to bulk samples. Another important point to outline is the increase of the glass contribution (halo around 20°) in the XRD pattern due to the presence of the cladding glass.

### FTIR

**Fig. 3:**
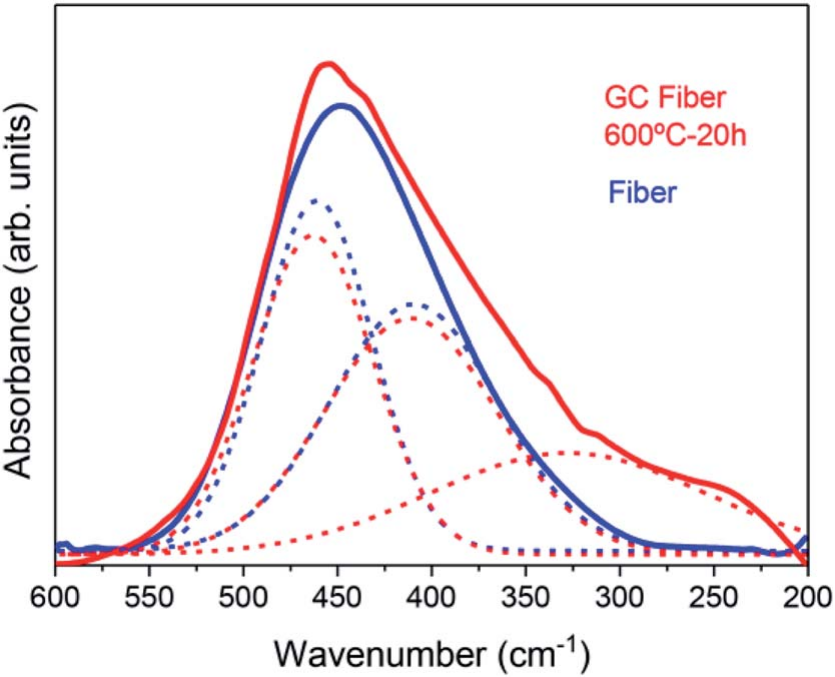
Comparison of the FTIR spectra of the GC fiber treated at 600 °C for 20 h and of the untreated fiber.

A further confirmation of NCs growth is obtained by FTIR spectra in the range 600–200 cm^−1^ given in Fig. 3. For the untreated fiber, the absorption band can be simply deconvoluted in two bands corresponding to Si–O–Si rocking vibrations and skeletal deformation of Si–O–Si links centered at ∼460 and 411 cm^−1^, respectively.^42,43^ For the GC fiber treated at 600 °C for 20 h, the same Si–O–Si vibrations are obtained but a further contribution, that can be represented by a Gaussian centered at ∼330 cm^−1^, broadens the spectrum and is associated to the growth of fluoride NaLuF_4_-type NCs.^44^ The relevant broadening of this band can be related to the nature of the NCs that is a cubic solid solution which most general formula is Na_*x*_Lu_2*x*−1_F_7*x*−3_.^15,38^

### HRTEM

**Fig. 4:**
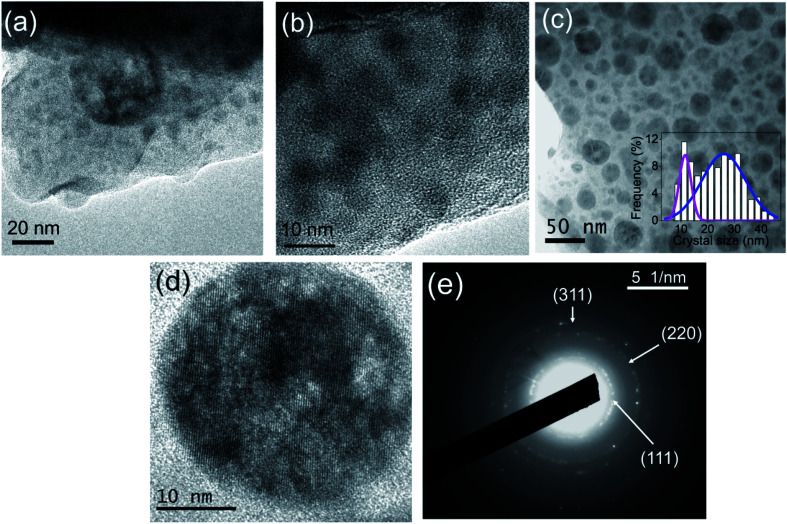
HRTEM micrograph of the (a and b) as made fiber and (c and d) GC fiber treated at 600 °C for 20 h. (e) SAED pattern of GC fiber treated at 600 °C for 20 h.

HRTEM micrographs of the as made fibers are shown in Fig. 4(a and b). Typical phase separation droplets are observed in agreement with the behavior of the bulk glass.^38^ After heat treatment at 600 °C for 20 h the presence of fluoride NCs is clearly observed, Fig. 4(c and d). A broad crystal size distribution is observed and the data fit well with two Gaussian functions centered at 15 and 23 nm. By a weighted average of the two contributions, an average crystal size ∼20 nm is obtained, confirming the XRD results. Fig. 4(e) shows the SAED pattern for the GC fiber treated at 600 °C for 20 h, confirming the presence of NCs. The analysis of the SAED pattern and its comparison with the JCPDS pattern of NaLuF_4_ has also allowed the assignment of the Miller indexes. The corresponding values for the (111), (220) and (311) planes are 0.30, 0.19 and 0.16 nm, respectively, in good agreement with the value reported for the NaYF_4_ but with smaller values due to the smaller size of Lu^3+^, Er^3+^ and Yb^3+^ with respect to Y^3+^.^45^

### Optical properties

#### Losses

The propagation losses of these multimode optical GC fibers codoped with 0.5% ErF_3_–2 mol% YbF_3_ are about 10 dB m^−1^. This value is similar to that obtained for different GC optical fibers.^28,29,46^

#### UC emission

Room temperature UC emission spectra were performed in GC optical fibers codoped with 0.5 mol% ErF_3_ and 2 mol%YbF_3_, by exciting at 980 nm in resonance with the ^4^I_11/2_ (Er^3+^) and ^2^F_5/2_ (Yb^3+^) levels. According to previous results obtained in bulk GC samples, this YbF_3_ concentration maximizes the overall Er^3+^ UC luminescence. Further addition of Yb^3+^ ions leads to no further increase in the Er^3+^ UC luminescence probably due to energy migration among Yb^3+^ ions and the presence of energy back transfer.^37^

**Fig. 5:**
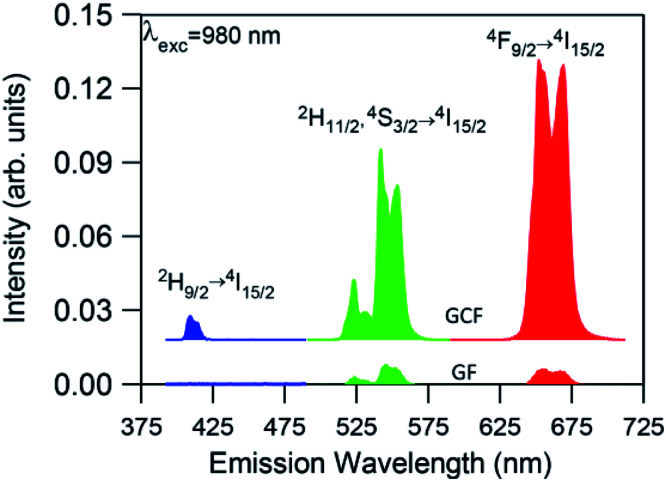
Room temperature UC emission spectra for the glass (GF) and glass-ceramics fiber (GCF) obtained under excitation at 980 nm with an excitation power density of 19 W cm^−2^. The fibers length was 2 cm.

The UC emission spectra of the GC fibers obtained under 980 nm excitation show the characteristic green emissions, attributed to the transitions from the two thermally ^2^H_11/2_ and ^4^S_3/2_ levels to the ground state, together with the red emission corresponding to the ^4^F_9/2_ → ^4^I_15/2_ transition. In addition, a weak blue emission is observed at around 410 nm which corresponds to the ^2^H_9/2_ → ^4^I_15/2_ transition. As an example Fig. 5 shows the UC emission spectrum for an excitation power density of 19 W cm^−2^. The UC emission spectrum of the glass fiber (GF), obtained under the same experimental conditions, has also been included for comparison. As can be observed, the spectrum of the GC fiber shows better resolved bands together with an enhanced intensity by more than one order of magnitude which suggests the incorporation of the rare-earth ions in the crystalline phase. A fluorine-rich environment around the rare-earth ions in the GC fiber, with lower maximum phonon energy than in the glass matrix, leads to a reduction in the vibration energy of the phonons coupled to Er^3+^ ions which benefits the UC emission. Thus, the incorporation of the RE ions in the fluoride NCs leads to a reduction of the multiphonon relaxation rates and to an increase of the quantum efficiency of emission of rare-earth ions. Moreover, the ratio between the red and green emission intensities increases from 0.95 in the glass fiber to 1.36 in the GC fiber. This enhancement of the red emission in the GC fiber, previously observed in other glass-ceramics, may be due to an increase of the energy transfer processes populating the ^4^F_9/2_ level. This behaviour could be associated to a higher concentration of rare-earth ions in the NCs, which reduces the ion–ion distances and increases the probability of energy transfer processes.^47,48^

The presence of the weak UC blue emission due to the ^2^H_9/2_ → ^4^I_15/2_ transition in the GC fiber is remarkable because it reveals the high efficiency of the GC fiber as this emission is generally not observed due to the low efficiency of the three or four photon UC processes.

**Fig. 6:**
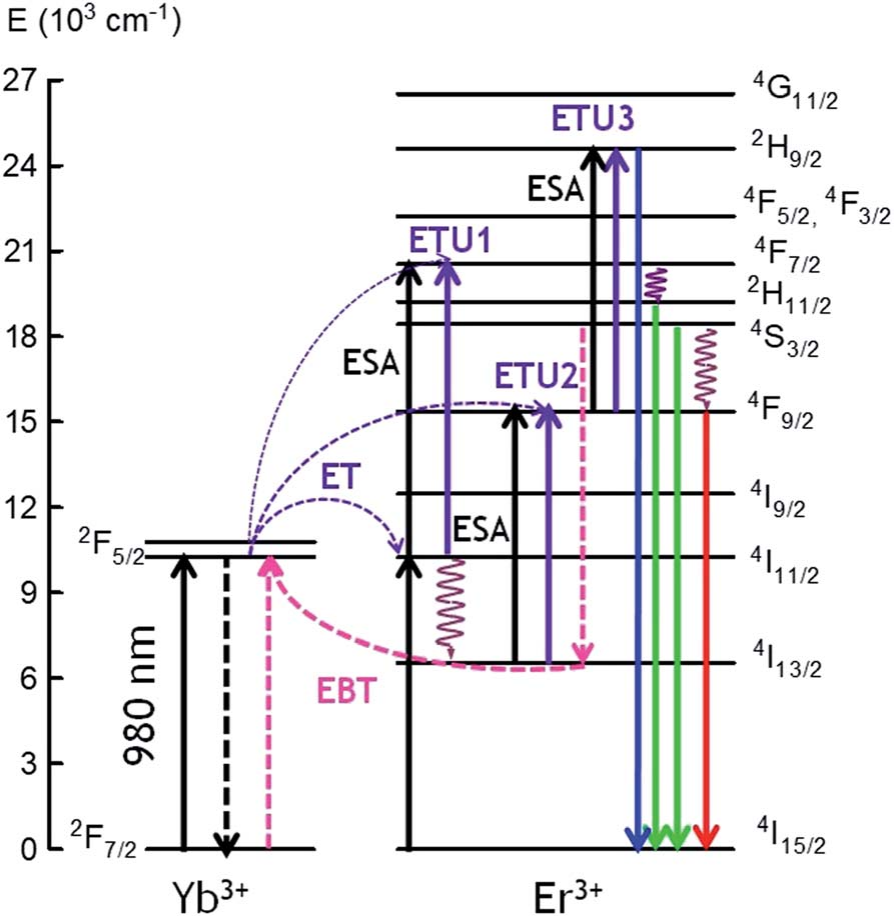
Simplified energy level diagram and possible populating pathways of codoped fibers under 980 nm excitation.

The possible UC mechanisms to account for the visible UC emissions under 980 nm excitation are shown in Fig. 6. First NIR laser photons directly excite the Yb^3+^ and Er^3+^ ions from the ground state to the ^2^F_5/2_ (Yb^3+^) and ^4^I_11/2_(Er^3+^) excited states by ground state absorption: (^2^F_7/2_ + photon → ^2^F_5/2_) (Yb^3+^) and (^4^I_15/2_ + photon → ^4^I_11/2_) (Er^3+^). Since the Yb^3+^ ions have larger absorption cross-section than the Er^3+^ ions at 980 nm, the population of the ^4^I_11/2_ mainly occurs by energy transfer from the Yb^3+^ ions through the (^2^F_5/2_ → ^2^F_7/2_) (Yb^3+^):(^4^I_15/2_ → ^4^I_11/2_) (Er^3+^) (ET) process. The Er^3+^ ions in the ^4^I_11/2_ level can be excited to the ^4^F_7/2_ state by excited state absorption (ESA) of a second infrared photon ^4^I_11/2_ (Er^3+^) + photon → ^4^F_7/2_ (Er^3+^) or by energy transfer from an Yb^3+^ ion (^2^F_5/2_ → ^2^F_7/2_) (Yb^3+^):(^4^I_11/2_ → ^4^F_7/2_) (Er^3+^) (ETU1). The green emitting levels ^2^H_11/2_ and ^4^S_3/2_ are populated by multiphonon relaxation from the upper level ^4^F_7/2_.

The red emission is strongly enhanced under 980 nm excitation, which means that other excitation processes, as well as multiphonon relaxation from the ^4^S_3/2_ level, populate the ^4^F_9/2_ level. This conclusion is confirmed by the visible emission spectrum obtained under 488 nm excitation, in which the main emission corresponds to the green one. The red emitting level ^4^F_9/2_ can be populated by an excited state absorption (ESA) from the ^4^I_13/2_ level populated from the ^4^I_11/2_ level or by an energy transfer process described by (^2^F_5/2_ → ^2^F_7/2_) (Yb^3+^):(^4^I_13/2_ → ^4^F_9/2_) (ETU2) (Er^3+^). As it was observed in the bulk samples, the presence of Yb^3+^ ions has a significant influence, not only on the overall UC emission intensity, but also on the luminescence color through increasing the red emission intensity relative to the green one. This behavior, previously observed in other co-doped systems, has been attributed to an increase of the population of the ^4^F_9/2_ and ^4^I_13/2_ levels due to energy back transfer from Er^3+^ to Yb^3+^ through the (^4^S_3/2_ → ^4^I_13/2_) (Er^3+^):(^2^F_7/2_ → ^2^F_5/2_) (Yb^3+^) cross-relaxation process.^49–51^ This process reduces the population of level ^4^S_3/2_ and consequently the green emission. At the same time, the energy transfer described by (^2^F_5/2_ → ^2^F_7/2_) (Yb^3+^):(^4^I_13/2_ → ^4^F_9/2_) (Er^3+^) (ETU2) populates the ^4^F_9/2_ level giving rise to an enhancement of the red emission. The presence of energy back transfer has been confirmed in the bulk samples by the lifetime decrease of the green emission from level ^4^S_3/2_ (Er^3+^) obtained under 486 nm excitation.^39^

Finally, the weak blue emission from ^2^H_9/2_ level should be due to a three-photon UC process. There are different UC processes to explain the population of this level. Firstly, an ESA process from the ^4^F_9/2_ level can occur, promoting the Er^3+^ ions to the ^2^H_9/2_ level or an energy transfer process described by (^2^F_5/2_ → ^2^F_7/2_) (Yb^3+^):(^4^F_9/2_ → ^2^H_9/2_) (Er^3+^) (ETU3). In this process, once the ^4^F_9/2_ is populated *via* ETU2, an Yb^3+^ ion also in the excited state transfers its energy to the Er^3+^ ion which is promoted to the ^2^H_9/2_ state being the excess of energy dissipated by the lattice.^52^ In addition, we can not exclude the possibility that the blue UC emission occurs *via* Er^3+^ ions since the blue emission has also been observed in single doped GC bulk samples.^15^ In this case, two Er^3+^ ions in the ^4^S_3/2_ level can interact through a nearly-resonant energy transfer process determined by the pair of transitions (^4^S_3/2_ → ^4^I_9/2_):(^4^S_3/2_ → ^2^H_9/2_). Another possibility is the quasi-resonant ETU mechanism (^4^S_3/2_ → ^4^I_11/2_):(^4^S_3/2_ → ^2^G_11/2_).^53^ Besides, the ^2^H_9/2_ can be populated through a process in which the Er^3+^ ions in the ^4^F_9/2_ level relax to the ^4^I_13/2_ level and can transfer part of their energy to those in the ^4^S_3/2_ level which are promoted to the ^4^G_9/2_ according to the pair of transitions (^4^F_9/2_ → ^4^I_13/2_):(^4^S_3/2_ → ^4^G_9/2_).^54^ Then, the ^2^H_9/2_ level is reached by multiphonon-relaxation from the upper levels.

#### Pump power dependence of UC luminescence

To further investigate the excitation mechanisms involved in the UC luminescence in the GC fibers after 980 nm excitation, the UC emission spectra has been obtained at different pump power densities.

**Fig. 7:**
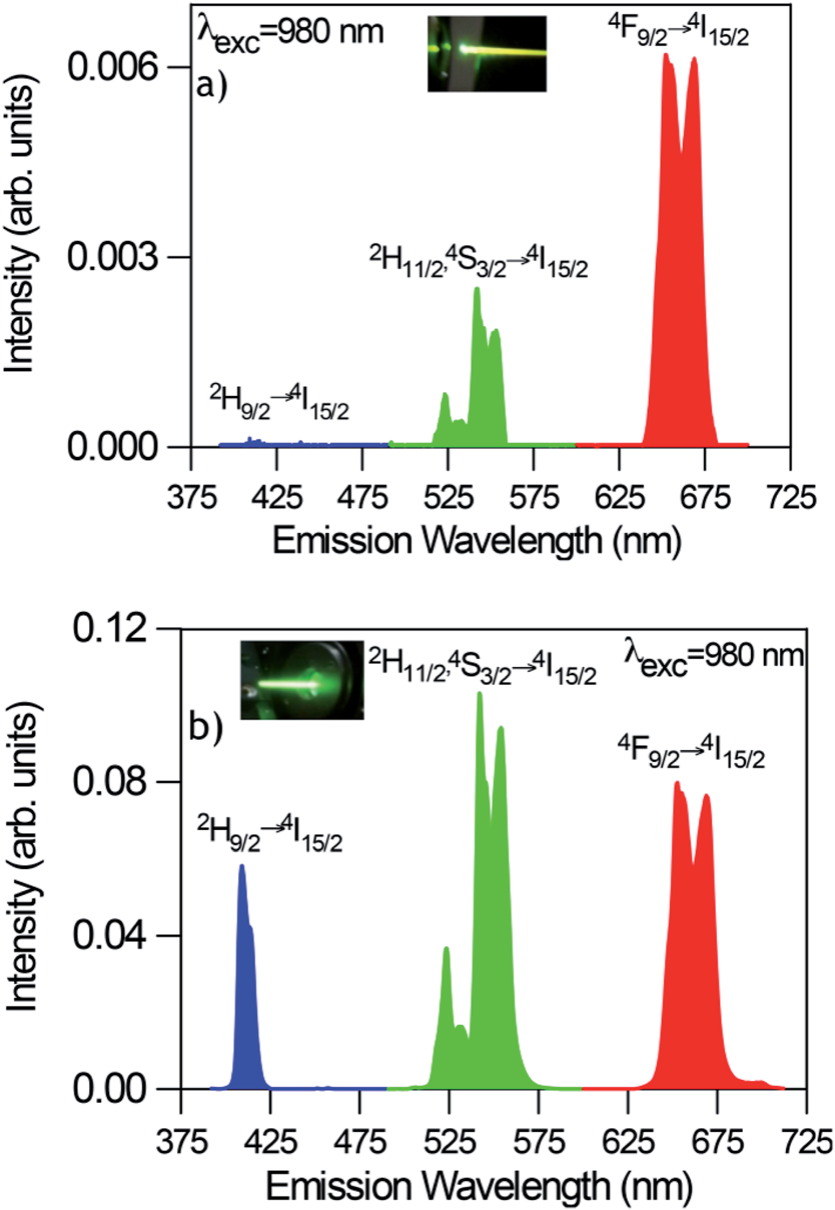
Room temperature UC emission spectra of GC fibers obtained under excitation at 980 nm at two different pump power densities (a) 2.5W cm^−2^ (unfocused) and (b) 0.75 kW cm^−2^ (focused).

**Fig. 8:**
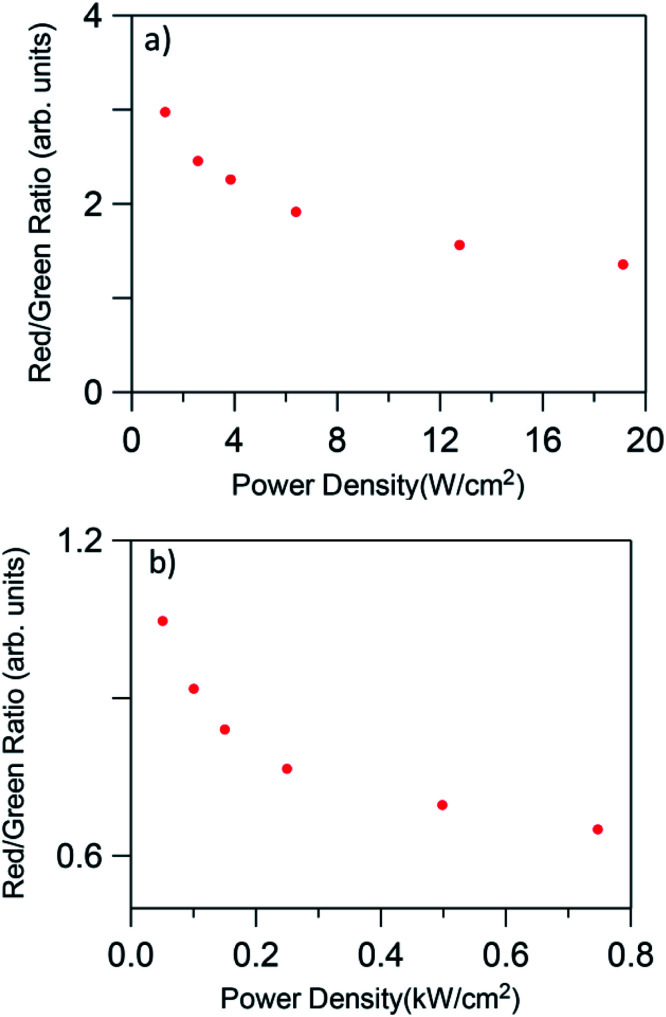
Red-to-green ratio dependence on pump power density for low (a) 1.3 to 19 W cm^−2^ (unfocused) and high (b) 0.025 to 0.75 kW cm^−2^ (focused) pumping regime.

In all cases, the spectra are characterized by the blue, green, and red emissions corresponding to the ^2^H_9/2_ → ^4^I_15/2_, (^2^H_11/2_,^4^S_3/2_) → ^4^I_15/2_, and ^4^F_9/2_ → ^4^I_15/2_ transitions respectively, but the relative intensity of the emissions is strongly dependent on the excitation power density. As an example Fig. 7 shows the UC emission spectra of the codoped GC fiber obtained at two different pump power densities, 2.5 W cm^−2^ (unfocused) and 0.75 kW cm^−2^ (focused). It is noticed that the UC emission exhibits significant changes in the red-to-green emission ratio and in the blue band intensity. As can be seen in the high power limit ≈ 0.75 kW cm^−2^, the blue emission increases significantly and the red-to-green ratio decreases. Fig. 8 shows the evolution of the red-to-green ratio for two different pumping regimes (a) from 1.3 to 19 W cm^−2^ (unfocused) and (b) 0.025 to 0.75 kW cm^−2^ (focused). The red-to-green ratio decreases from ≈3 to 0.65 when the pump power density changes from 1.3 W cm^−2^ to 0.75 kW cm^−2^ which indicates that the emission color changes from yellow to green. The color change is easily detected with the naked eye. A similar behavior is observed in bulk GC samples codoped with 0.5 mol% ErF_3_–2 mol% YbF_3_ in which the red-to-green ratio decreases from ≈3.5 to 0.79 when the pump power density changes from 1.3 W cm^−2^ to 0.75 kW cm^−2^.

**Fig. 9:**
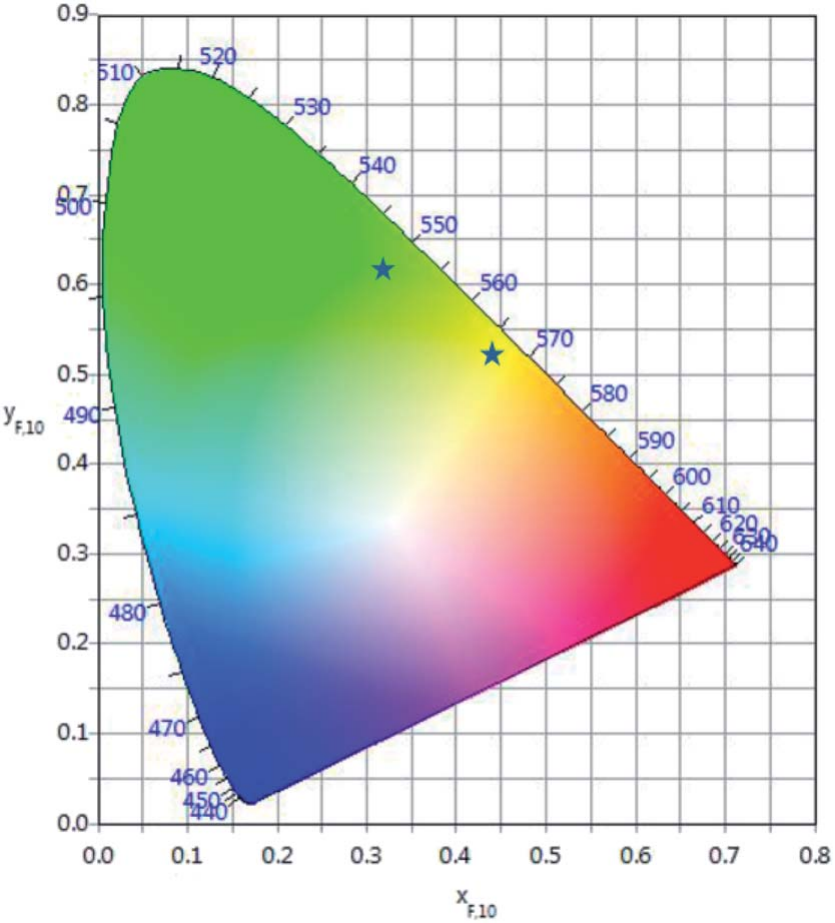
CIE chromaticity coordinates of the GC fiber for two different laser excitation densities.

The calculated chromaticity coordinates calculated for two laser power densities, 2.5 W cm^−2^ and 0.75 kW cm^−2^, are illustrated in Fig. 9. The stars represent the CIE coordinates. The chromaticity coordinates move from yellow (*x* = 0.4458, *y* = 0.5031) to green (*x* = 0.3361, *y* = 0.6097) when the laser power density increases from 2.5 W cm^−2^ to 0.75 kW cm^−2^ which means that the output color can be tuned by controlling the excitation power density.

The upconverted emissions did not show any remarkable thermal load effect induced by the pump laser at 980 nm within the power range utilized either in bulk or in the GC fiber as evidenced by a nearly constant (^2^H_11/2_ → ^4^I_15/2_)/(^4^S_3/2_ → ^4^I_15/2_) fluorescence intensity ratio. This behavior agrees with those found in other rare-earth-doped glass-ceramics pumped with power densities of the same order.^17^

To obtain more information about the UC processes, the dependence of the green and red UC emissions has been obtained as a function of the incident pump power density. It is well known that the UC emission intensity (*I*_up_) depends on the incident pump power (*P*_pump_) according to the relation *I*_up_ ∝ (*P*_pump_)^*n*^, where *n* is the number of photons involved in the pumping mechanism. When UC emission is excited by sequential absorption and energy transfer UC of *n* photons, its dependence on the incident pump power *P*_pump_ decreases from (*P*_pump_)^*n*^ to *P*_pump_ as long as the UC rate exceeds the decay rate from the intermediate states.^55,56^

**Fig. 10:**
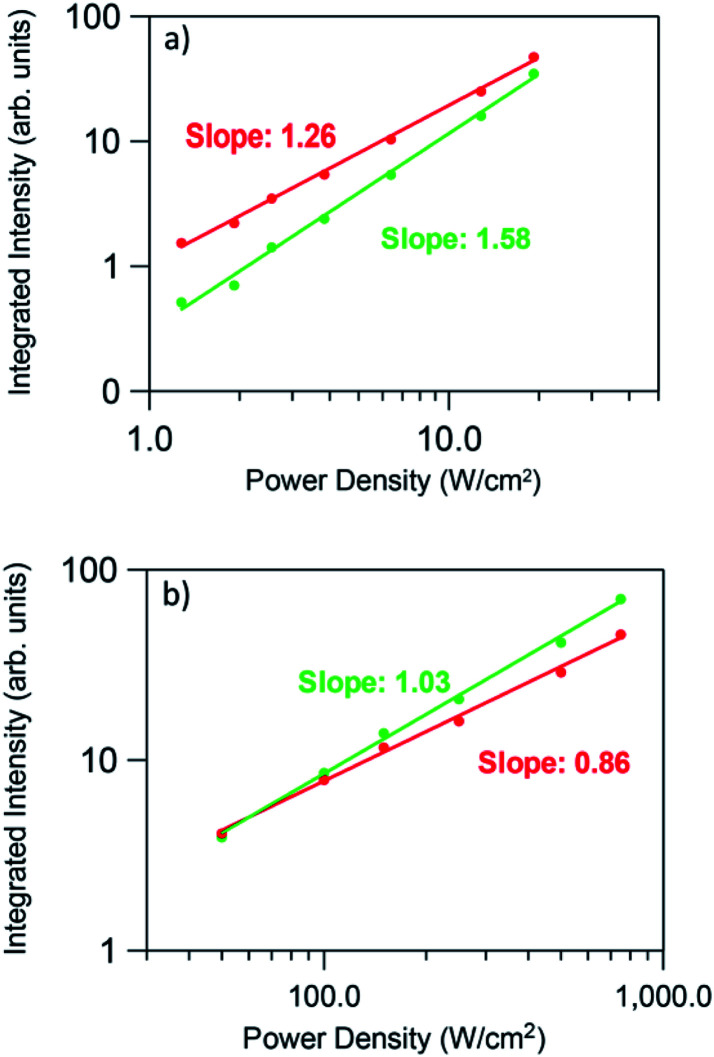
Dependence of the green and red integrated emission intensities on the excitation power density (a) low pumping regime (1.3 to 19 W cm^−2^ unfocused) and (b) high pumping regime (0.025 to 0.75 kW cm^−2^ focused).

**Fig. 11:**
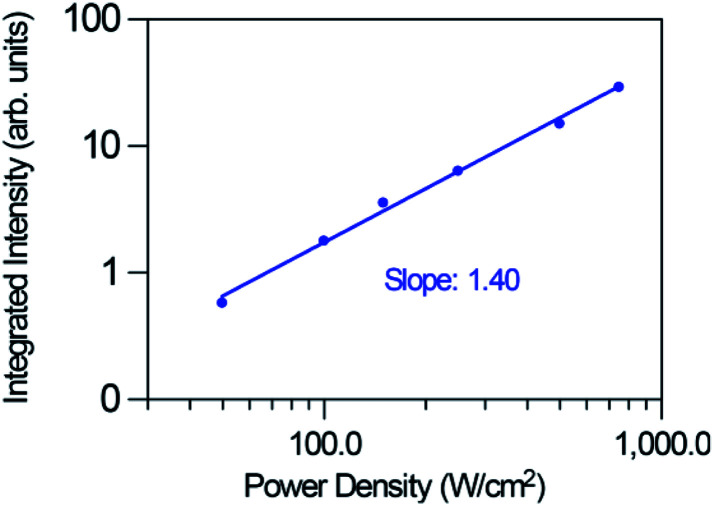
Dependence of the blue upconversion emission intensity on the excitation power density for the high pumping regime (0.025 to 0.75 kW cm^−2^ focused).

Fig. 10 shows the logarithmic plot of the UC green and red emission intensities of the co-doped GC fiber as a function of the pump power laser density for two different pumping regimes (1.3 to 19 W cm^−2^) and (0.025 to 0.75 kW cm^−2^). As can be seen for the red emission, the slope decreases from 1.26 to 0.86 as the pump power density increases, which indicates that only one photon is required to excite the electrons to the ^4^F_9/2_ state. However, it is clear from the energy level diagram of Er^3+^ ions that two photons are needed to populate this level. A similar behavior is observed for the green emission for which the slope decreases from 1.58 to 1.03. In the case of the blue emission attributed to the ^2^H_9/2_ → ^4^I_15/2_ transition, according to the energy level diagram, this emission should be due to a three-photon UC process. However, as can be seen in Fig. 11, the pump power dependence of this emission measured in the high pumping regime between 0.025 and 0.75 kW cm^−2^ (the blue emission can be accurately measured only in the high pumping regime), gives a slope of 1.40.

This behavior, previously observed in other systems has been attributed to the competition between the decay rate of the intermediate state and the UC rates. This effect was theoretically described by Pollnau *et al.*^55^ The model predicts that the UC luminescence from a state that requires *n* excitation photons will have a slope of *n* in the low-power regime when the luminescence intensity is plotted in a double logarithmic representation *versus* absorbed pump intensity. Higher pump powers and consequently increasing competition between linear decay and UC for the depletion of the intermediate excited state results in a reduced slope. It was experimentally observed that when UC dominates over linear decay for the depletion of the intermediate excited state, the slope of the luminescence from the upper state *n* is almost linear.^55^ As suggested by Suyver *et al.*,^56^ this saturation effect proves that in these GC fibers a sensitized energy transfer upconversion process is responsible for populating the emitting levels of Er^3+^.

## Conclusions

Transparent glass-ceramics were successfully obtained by the rod-in-tube method and subsequent crystallization process. Cubic NaLuF_4_ nanocrystals were obtained after the heat treatment at 600 °C, being the average crystal size around 20 nm. Intense green and red upconversion emissions due to (^2^H_11/2_,^4^S_3/2_) → ^4^I_15/2_ and ^4^F_9/2_ → ^4^I_15/2_ transitions respectively together with a more weak blue emission due to ^2^H_9/2_ → ^4^I_15/2_ transition of Er^3+^ ions has been observed under excitation at 980 nm in sodium lutetium fluoride glass-ceramic optical fibers codoped with Yb^3+^ ions. The intensity of the green and red upconversion bands shows a nearly linear dependence on the excitation power which can be explained by saturation effects in the intermediate energy states. The upconversion emission color changes from yellow to green by increasing the excitation power density which allows the color output of the Er^3+^ emission to be tuned. Increasing the excitation power density from 1.3 W cm^−2^ to 0.75 kW cm^−2^, the red-to-green ratio decreases from ≈3 to 0.65, which indicates that the emission color changes from yellow to green as can be easily recognized by naked eyes. The observed behavior makes these optical glass-ceramic fibers promising candidates for photonic applications.

## Conflicts of interest

There are no conflicts to declare.

## Supplementary Material
